# The Influence of Body Contouring Surgery on Weight Control and Comorbidities in Patients After Bariatric Surgery

**DOI:** 10.1007/s11695-019-04298-1

**Published:** 2019-12-02

**Authors:** C. E. E. de Vries, M. C. Kalff, E. M. van Praag, J. M. G. Florisson, M. J. P. F. Ritt, R. N. van Veen, S. M. M. de Castro

**Affiliations:** 1 Department of Surgery, OLVG, Amsterdam, The Netherlands; 2 Obesity Center Amsterdam, OLVG West, Amsterdam, The Netherlands; 3 Department of Plastic, Reconstructive and Hand Surgery, OLVG, Amsterdam, The Netherlands; 4 Department of Plastic, Reconstructive and Hand Surgery, Amsterdam UMC, location VUmc, Amsterdam, The Netherlands

**Keywords:** Bariatric surgery, Body contouring surgery, Weight loss, Comorbidities

## Abstract

**Introduction:**

A considerable number of patients experience some long-term weight regain after bariatric surgery. Body contouring surgery (BCS) is thought to strengthen post-bariatric surgery patients in their weight control and maintenance of achieved improvements in comorbidities.

**Objectives:**

To examine the impact of BCS on long-term weight control and comorbidities after bariatric surgery.

**Methods:**

We performed a retrospective study in a prospective database. All patients who underwent primary Roux-en-Y gastric bypass (RYGB) and presented for preoperative consultation of BCS in the same hospital were included in the study. Linear and logistic mixed-effect model analyses were used to evaluate the longitudinal relationships between patients who were accepted or rejected for BCS and their weight loss outcomes or changes in comorbidities.

**Results:**

Of the 1150 patients who underwent primary RYGB between January 2010 and December 2014, 258 patients (22.4%) presented for preoperative consultation of BCS. Of these patients, 126 patients eventually underwent BCS (48.8%). Patients who were accepted for BCS demonstrated significant better ∆body mass index (BMI) on average over time (− 1.31 kg/m^2^/year, 95% confidence interval (CI) −2.52 − −0.10, *p* = 0.034) and percent total weight loss (%TWL) was significantly different at 36 months (5.79, 95%CI 1.22 – 10.37, *p* = 0.013) and 48 months (6.78, 95%CI 0.93 – 12.63, *p* = 0.023) after body contouring consultation. Patients who were accepted or rejected did not differ significantly in the maintenance of achieved improvements in comorbidities.

**Conclusion:**

BCS could not be associated with the maintenance of achieved improvements in comorbidities after bariatric surgery, whereas it could be associated with improved weight loss maintenance at 36 and 48 months after body contouring consultation. This association should be further explored in a large longitudinal study.

## Introduction

Severe obesity has become a major health concern over the last decades [1]. Bariatric surgery is considered to be the most effective treatment resulting in significant weight loss, remission of obesity-related comorbidities, and substantial improvement in health-related quality of life (HRQoL) [2–7]. A considerable number of patients fail to achieve sufficient weight loss or even experience significant weight regain after initial weight loss [8, 9]. Inadequate weight control is associated with recurrence of obesity-related comorbidities and recurrent pharmaceutical costs [10]. As weight regain negatively impacts patient health and long-term healthcare costs, preservation of weight loss is of utmost importance.

Substantial weight loss following bariatric surgery has led to a new population of body-contouring patients with deformities spanning all regions of the body [11]. Excess skin after bariatric surgery appears to be very common and causes psychosocial and functional impairment [12–14]. Body contouring surgery (BCS) reduces excess loose skin and has been demonstrated to improve daily impairments associated with excess skin and might therefore strengthen post-bariatric surgery patients in their weight control [15–20]. Previous studies described that BCS holds promise in optimizing weight control after bariatric surgery [21–25]. However, none of these studies reported on the recurrence or worsening of comorbidities after BCS in post-bariatric patients.

Many post-bariatric patients seek consultation concerning BCS, but not all patients undergo these procedures [11, 26–29]. This could be due to rejection by either the plastic surgeon or more commonly the healthcare insurer. The major barrier to undergoing BCS is financial [30]. Taking the high costs of weight regain into account, it should be noted that BCS, conversely, may have the potential to influence post-bariatric care costs in the healthcare system. This study will examine the impact of BCS on long-term weight control and comorbidities after bariatric surgery.

## Methods

### Study Design and Population

This is a retrospective study using a consecutive electronic database of patients who underwent bariatric surgery from January 2010 until December 2014 in the Obesity Center Amsterdam, OLVG, The Netherlands. Only patients who underwent primary Roux-en-Y gastric bypass (RYGB) were included in the study. Other surgical procedures and revisional procedures were excluded from this study to avoid bias due to different weight loss outcomes associated with the different procedures. Patients were included in the study if they consulted the plastic surgery department for BCS following primary RYGB in the same hospital. Patients were subdivided into two study groups: (1) patients who were accepted for BCS and (2) patients who were rejected for BCS. All body contouring procedures were covered by healthcare insurer. In The Netherlands, all healthcare insurers follow the same national guidelines to decide whether or not to cover a body contouring procedure: (1) The patient must have had bariatric surgery more than 18 months before body contouring surgery. (2) The patient should have a stable weight for more than 12 months. (3) The patient’s body mass index (BMI) should be less than 30 or 35 kg/m^2^ (dependent on the procedure). (4) The excess skin of the patient should be “a disfigurement” or “a demonstrable physical dysfunction.” All patients who underwent bariatric surgery at the Obesity Center Amsterdam met the International Federation for the Surgery of Obesity and Metabolic Disorders criteria for bariatric surgery [31]. The Institutional Medical Ethics Committee provided approval for this study and informed consent was not necessary for this retrospective study.

### Data Collection

The data were collected from electronic medical records, deidentified and entered into a database. Collected patient variables include gender, age, height, Pittsburgh Rating Scale (PRS) classification (used to classify for the amount of excess skin, range 0 (indicating normal appearance) to 3 (indicating the most severe form of excess skin)), and the presence of type 2 diabetes (T2D), hypertension, dyslipidemia, and obstructive sleep apnea (OSA) at body-contouring consultation. Body contouring procedures included abdominoplasty, brachioplasty, and mastopexy with breast reduction or augmentation, buttock lift, and thigh lift. If available, the weight of resected tissue was recorded. Body weight and comorbidities were assessed pre-bariatric surgery, at body contouring consultation and 6, 12, 24, 36, 48, and 60 months after body contouring consultation (if available). Weight loss was calculated as stated in the most recent guidelines: BMI in kg/m^2^, percent excess weight loss (%EWL), and percent total weight loss (%TWL) [32]. Weight change was calculated as ∆BMI (Δkg/m^2^ per year). Weight change was calculated from the first body contouring procedure in case of more than one procedure.

### Statistical Analysis

Patient characteristics were described as the mean ± standard deviation (SD) or by percentages. Depending on the distribution of the data (normality) evaluated by histograms, continuous baseline variables were compared with independent *t* test or Mann-Whitney *U* test and categorical variables were compared with chi-square test. Descriptive analyses were performed using SPSS 22.0 for Windows (SPSS Inc., Chicago, IL, USA). The longitudinal relationships between patients who were accepted or rejected for BCS and weight loss outcomes (∆BMI and %TWL) were analyzed using linear mixed models, taking into account a two-level structure: repeated measures of body weight were clustered within patients. Before analysis, normality of weight, BMI, %TWL, and Apnoea–Hypopnoea Index (AHI) (indicating OSA) were assessed with histograms. Logistic mixed model analyses were performed to assess the longitudinal association between patients who were accepted or rejected for BCS and change in comorbidities (T2D, dyslipidemia, hypertension, and OSA). We evaluated whether all assumptions for regression analysis were met for further analyses. In addition to the crude analyses, we performed linear mixed model analyses adjusted for sex, age, PRS classification, and baseline body weight or BMI in case of confounding. To detect effect modification of undergoing BCS, further analyses were performed with the interaction term whether or not a patient was accepted or rejected for BCS. Afterward, time and the interaction between time and group (whether or not a patient was accepted for BCS) were added to the adjusted mixed model to investigate whether the intervention effect (BCS) differed across time points (a *p* value < 0.05 indicated an interaction effect). Additional analyses were performed to assess effect modification by the number of body contouring procedures (i.e., whether the effect of body contouring surgery on weight control was different for the number of body contouring procedures) and abdominoplasty versus other body contouring procedures (i.e., whether the effect of body contouring surgery on weight control was different for abdominoplasty versus other body contouring procedures). The mixed model analyses were performed using Stata 14 software (StataCorp. 2015. Stata Statistical Software: Release 14. College Station, TX: StataCorp LP). Findings were considered statistically significant for a two-tailed significance level of *p* < 0.05.

## Results

### Study Population

Overall, 1150 patients underwent primary RYGB from January 2010 until December 2014 and 258 patients (22.4%) presented for consultation for BCS. Patient characteristics (gender, age, and BMI) did not differ significantly between patients who did or did not present for body contouring consultation. Of the patients who presented for body contouring consultation, 126 patients were accepted and underwent BCS (48.8%). Of the 132 patients who were rejected and did not receive BCS, 23 patients (17.4%) were rejected by the plastic surgeon. Reasons for rejection by the plastic surgeon were a high BMI (17 patients), unstable weight (three patients), and smoking habits (three patients). The major other reason that patients were rejected for surgery was the lack of insurance coverage (109 patients (82.6%)). Reasons for rejection by the insurer were a high BMI (13 patients), unstable weight (1 patient), the lack of functional problems (34 patients), disagreement of the insurance company on the PRS classification (19 patients). Two patients canceled their procedure. The actual reason was unclear or inconclusive for the other 40 patients who were rejected by healthcare insurer.

Of the total study population, 225 patients (87.2%) were female. Mean age was 43.8 years (± 10.5), mean BMI pre-bariatric surgery was 45.4 kg/m^2^ (± 6.6), and mean BMI at body contouring consultation was 29.8 kg/m^2^ (± 4.8). Mean percentage excess weight loss (%EWL) was 78.5 (19.6) and mean percentage total weight loss (%TWL) was 33.7 (± 8.1) from bariatric surgery to body contouring consultation. The mean time between bariatric surgery and body contouring consultation was 22.5 months (± 9.9). Patient characteristics are shown in Table 1 for both the total study population and the two study groups: patients who were accepted or rejected for BCS. Both groups differed significantly for BMI and PRS classification at preoperative body contouring consultation, but did not differ for gender, age, %EWL, %TWL, and comorbidities.

**Table 1:** Baseline variables between patients who were accepted or rejected for body contouring surgery

	Total (*N* = 258)	Accepted for BCS (*N* = 126)	Rejected for BCS (*N* = 132)	*p* value
Gender; female; *n* (%)	225 (87.2)	112 (90)	111 (85)	0.310‡
Age; years (±)	43.8 (10.5)	44 (10.3)	43 (10.7)	0.442†
BMI; kg/m^2^ (±) (pre-bariatric surgery)	45.4 (6.6)	44.8 (6.1)	45.8 (7.2)	0.222†
BMI; kg/m^2^ (±) (BCS consultation)	29.8 (4.8)	29.1 (3.7)	30.6 (5.7)	0.012†*
%EWL (±) from bariatric surgery to body contouring consultation	78.5 (19.6)	80.1 (17.5)	76.8 (21.4)	0.190†
%TWL (±) from bariatric surgery to body contouring consultation	33.7 (8.1)	34.4 (8.5)	33.1 (7.8)	0.203†
PRS 0 = normal appearance 1 = a mild form of excess skin 2 = moderate form of excess skin 3 = the most severe form of excess skin	0) 0 1) 6 2) 48 3) 82	0) 0 1) 3 2) 15 3) 55	0) 0 1) 3 2) 33 3) 27	< 0.001‡*
Type 2 diabetes; *n* (%)	83 (33)	47 (38)	36 (28)	0.091‡
Hypertension; *n* (%)	114 (45)	61 (49)	53 (41)	0.217‡
Dyslipidemia; *n* (%)	30 (13)	19 (17)	11 (10)	0.094‡
OSA; *n* (%)	153 (60)	68 (55)	82 (64)	0.136‡

± standard deviation; *PRS*, Pittsburgh Rating Scale; *BCS*, body contouring surgery; *BMI*, body mass index; *OSA,* obstructive sleep apnea; *%TWL*, percentage total weight loss

‡*χ*^2^ test

†Independent *t* test

*Significant difference between patient groups (BCS or no BCS), *p <* 0.05

The most prevalent procedure was abdominoplasty (64%) and the buttock lift was only performed in one patient (< 1%) (Table 2). The weight of removed tissue during the body contouring procedure was available in 146 procedures. The median weight of the resected tissue ranged from 0.3–2.4 kg (minimum 0.1, maximum 15) (Table 2). Ninety-two patients underwent one body contouring procedure, 30 patients underwent two body contouring procedures, and four patients underwent three procedures.

**Table 2:** The different body contouring procedures and tissue weight removed

	Body contouring procedures (*N* = 164 in 126 patients) (*N* (%))	Tissue weight removed (kg) (median (range))
Abdominoplasty	105 (64)	2.4 (0.4 – 15)
Brachioplasty	9 (5)	0.3 (0.2 – 0.4)
Mastopexy	35 (21)	0.5 (0.1 – 2.8)
Buttock lift	1 (< 1)	Not available
Thigh lift	12 (7)	0.3 (0.1 – 1.5)

### Weight Loss

**Fig. 1:**
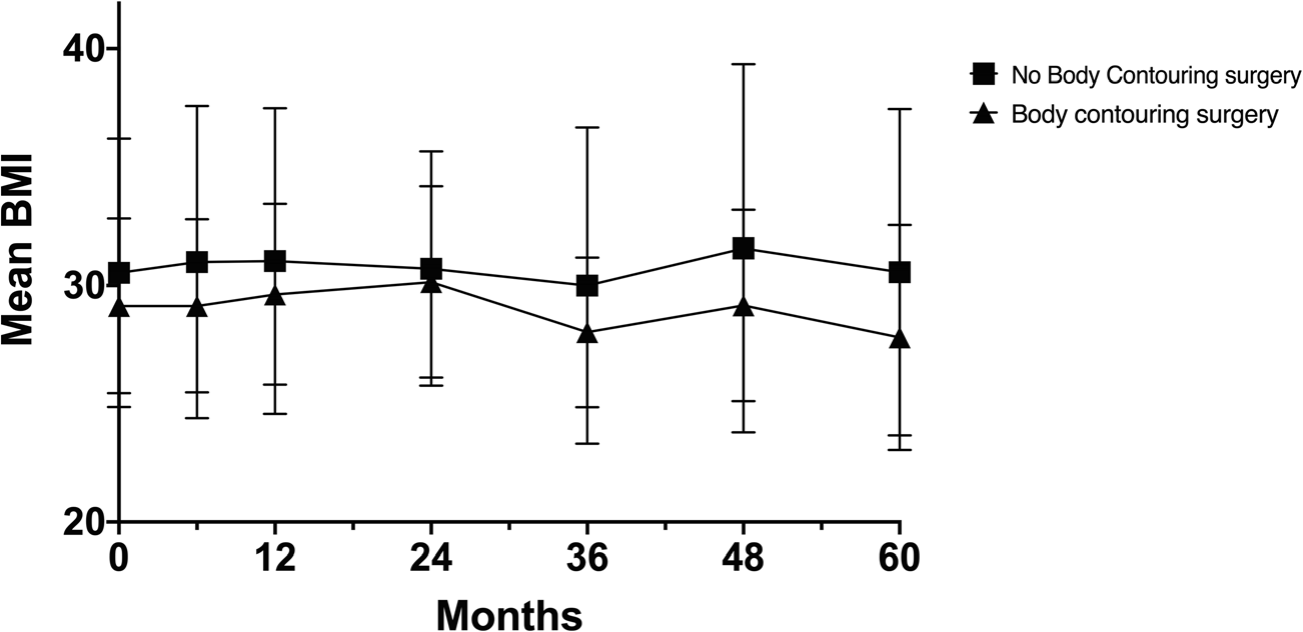
Mean ∆BMI over the different follow-up moments for patients who were accepted or rejected for body contouring surgery after body contouring consultation (corrected for the weight of tissue removed and adjusted for PRS classification and preoperative/body contouring consult BMI)

Patients who were accepted for BCS after bariatric surgery demonstrated significant better ∆BMI (adjusted: mean difference −1.31 kg/m^2^/year, 95% confidence interval (CI) − 2.52 – −0.10, *p* = 0.034 (unadjusted: mean difference −1.96 kg/m^2^/year , 95%CI −3.53 – 0.40, *p* = 0.040)) on average over time (over a period of 60 months) corrected for the weight of tissue removed and adjusted for PRS classification and preoperative/body contouring consult BMI compared to patients who were rejected for BCS after bariatric surgery. The differences between the patient groups were statistically significant at 36 months (mean difference −2.03, 95%CI −3.56 – −0.49, *p* = 0.010) and 48 months (mean difference − 2.71, 95%CI −4.65 – −0.76, *p* = 0.006) after body contouring consultation when investigating whether the intervention effect (BCS) differed across time points (Table 3, Fig. 1). ∆BMI was not significantly different for the number of body contouring procedures that patients underwent. The effect of body contouring surgery on ∆BMI was not significantly different between abdominoplasty and other body contouring procedures.

**Table 3:** Linear mixed models showing the relationship between patients who were accepted or rejected for body contouring surgery and ∆BMI and %TWL

	Follow-up	No. of participants	Coefficient	*p* value	95% confidence intervals	
∆BMI (kg/m^2^/year) (mean difference −1.31 kg/m^2^/year, 95%CI −2.52 – −0.10, *p* = 0.034*)	12-month	193	− 0.75	0.170	− 1.82	0.32
	24-month	111	− 0.59	0.391	− 1.94	0.76
	36-month	94	− 2.03	0.010*	− 3.56	− 0.49
	48-month	74	− 2.71	0.006*	− 4.65	− 0.76
	60-month	59	− 3.05	0.163	− 7.33	1.23
%TWL (%/year) (mean difference 0.65 %/year, 95%CI −2.37 – 3.67, *p* = 0.673)	12-month	193	2.20	0.174	− 0.97	5.37
	24-month	111	1.51	0.465	− 2.54	5.56
	36-month	94	5.79	0.013*	1.22	10.37
	48-month	74	6.78	0.023*	0.93	12.63
	60-month	59	8.70	0.192	− 4.38	21.78

†Difference in ∆BMI and %TWL between patient groups in relation to baseline (body contouring consultation)) corrected for the weight of tissue removed and adjusted for PRS classification and preoperative/body contouring consult BMI

*Significant difference in ∆BMI and %TWL between patient groups in relation to baseline, *p <* 0.05

**Fig. 2:**
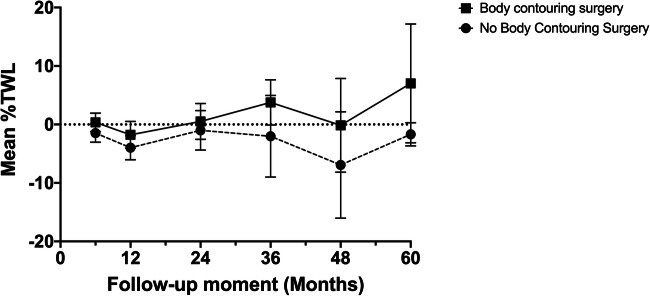
Mean %TWL over the different follow-up moments for patients who were accepted or rejected for body contouring surgery after body contouring consultation (corrected for the weight of tissue removed and adjusted for PRS classification and preoperative/body contouring consult BMI)

The %TWL between these patient groups was significantly different at 36 months (mean difference 5.79, 95%CI 1.22 – 10.37, *p* = 0.013) and 48 months (mean difference 6.78, 95%CI 0.93 – 12.63, *p* = 0.023) after body contouring consultation in relation to preoperative consultation (Table 3). At 36 months after body contouring consultation, patients who were accepted for BCS had increased weight loss, while patients who were rejected for BCS suffered from weight regain (Fig. 2). On average over time (over a period of 60 months), however, patients who were accepted for BCS after bariatric surgery did not differ significantly in %TWL (adjusted: mean difference 0.65 %/year, 95%CI −2.37 – 3.67, *p* = 0.673) (unadjusted: mean difference 0.89 %/year, 95%CI −2.21 – 4.00, *p* = 0.572)) corrected for the weight of tissue removed and adjusted for PRS classification and preoperative/body contouring consult BMI compared to patients who were rejected for BCS after bariatric surgery. %TWL did not differ significantly between the two patient groups at other follow-up moments. %TWL was not significantly different for the number of body contouring procedures that patients underwent. The effect of body contouring surgery on %TWL was not significantly different between patients undergoing abdominoplasty and patients undergoing other body contouring procedures.

### Comorbidities

Patients who were accepted for BCS did not differ significantly from patients who were rejected for BCS in the conservation of achieved improvements in the T2D (odds ratio (OR) 0.47, 95%CI 0.08 − 2.79, *p* = 0.406) and hypertension (OR 0.26, 95%CI 0.05 – 1.40, *p* = 0.116). Not enough follow-up data (*N* < 10) was available to perform the analysis to assess the longitudinal association between patients who were accepted or rejected for BCS and AHI (indicating OSA) or dyslipidemia.

## Discussion

The present study examined the difference in long-term weight loss outcomes and conservation of achieved improvements in comorbidities between patients accepted or rejected for BCS after consultation with the plastic surgeon. In our study, only fifty percent of the patients who desired BCS received one or more body contouring procedure. The major reason for not being accepted for surgery was the lack of reimbursement by healthcare insurer. This study demonstrated that undergoing BCS after bariatric surgery may positively influence weight control. The patients who presented for body contouring consultation had good and comparable weight loss outcomes following bariatric surgery. Patients who were accepted for BCS continued this trend with positive %TWL (weight loss) over the years, while patients who were rejected for BCS showed negative %TWL (weight regain) over the years. Although the differences between these patient groups were small and not significant at all follow-up moments, these results suggest that undergoing BCS could enhance weight loss maintenance. These findings are in line with previous studies concluding that post-bariatric body contouring could be associated with a weight loss benefit [21–25].

Even though we recognize that underlying factors of weight regain are multifactorial, BCS may play a role in weight control in the long term for patients who present for BCS following bariatric surgery. BCS has been demonstrated to improve patients’ body image and HRQoL, which may result in an enhanced ability to maintain or even improve weight loss [15–19]. Improvement in patient satisfaction and body image has been associated with greater motivation to get a desired appearance and for this reason ought to have better outcomes on total weight loss [33]. Furthermore, the removed excess skin after BCS improves physical functioning, which could influence weight loss outcomes by exercise [20].

This is the first study that assessed the influence of BCS on conservation of achieved improvements in comorbidities after bariatric surgery. Better weight loss after BCS or prolonged weight maintenance probably results in better maintenance of the achieved improvements in comorbidities. In this study, we could not demonstrate that the resolution of comorbidities was significantly more often maintained following BCS. The amount of weight loss that post-bariatric patients achieved after BCS in this study may be too little to influence comorbidities. Moreover, the number of patients with comorbidities in our study was too small to reach sufficient statistical power for the analyses.

The present study has several limitations. First of all, this is a retrospective study in a single institution. Patients who went to other hospitals for BCS were excluded from analysis and private pay patients were not included in this study. The patient groups (accepted or rejected for BCS) did differ in BMI and PRS classification at body contouring consultation and we adjusted for these confounders in the analyses, but in this study, we could not investigate whether our results are influenced by other confounding factors such as physical activity and eating habits. This could introduce bias that could influence the measured association of undergoing BCS and weight loss outcomes. It is possible that the better candidates (not based on the official criteria of the plastic surgeon or healthcare insurer) were selected for BCS and hence have better weight loss outcome. Even though we adjusted for BMI and PRS, other factors could influence patient selection during consultation with the plastic surgeon or judgement by healthcare insurer and thus influence outcome. In addition, as a limitation of our retrospective design, we could not relate weight loss outcomes to HRQoL data. It would be interesting to evaluate weight loss outcomes and HRQoL in a larger cohort to increase insight into the relationship between these outcomes. The BODY-Q, a comprehensive and validated patient-reported outcome measure for weight loss and body contouring patients, could be used for this purpose [13, 34, 35].

A large longitudinal study should be undertaken to further explore the association between BCS and improved weight loss maintenance after bariatric surgery that has been demonstrated in the present study. Future research on this topic should also be undertaken to further investigate whether the rejection of BCS could influence the conservation of comorbidities and whether this could impact medication use for comorbidities.

## Conclusions

BCS could not be associated with the maintenance of achieved improvements in comorbidities after bariatric surgery, whereas it could be associated with improved weight loss maintenance at 36 and 48 months after body contouring consultation. This association should be further explored in a large longitudinal study.
